# Transgene expression knock-down in recombinant Modified Vaccinia virus Ankara vectors improves genetic stability and sustained transgene maintenance across multiple passages

**DOI:** 10.3389/fimmu.2024.1338492

**Published:** 2024-02-06

**Authors:** Patrick Neckermann, Madlen Mohr, Martina Billmeier, Alexander Karlas, Ditte R. Boilesen, Christian Thirion, Peter J. Holst, Ingo Jordan, Volker Sandig, Benedikt Asbach, Ralf Wagner

**Affiliations:** ^1^ Institute of Medical Microbiology and Hygiene, Molecular Microbiology (Virology), University of Regensburg, Regensburg, Germany; ^2^ ProBioGen AG, Berlin, Germany; ^3^ Department of Immunology and Microbiology, Center for Medical Parasitology, The Panum Institute, University of Copenhagen, Copenhagen, Denmark; ^4^ InProTher APS, Copenhagen, Denmark; ^5^ SIRION Biotech GmbH, Planegg-Martinsried, Germany; ^6^ Institue of Clinical Microbiology and Hygiene, University Hospital Regensburg, Regensburg, Germany

**Keywords:** MVA, poxviral vectors, Modified Vaccinia Ankara, transgene stability, transgene knock-down, viral vaccines, vaccine manufacturing, transgene maintenance

## Abstract

Modified vaccinia virus Ankara is a versatile vaccine vector, well suited for transgene delivery, with an excellent safety profile. However, certain transgenes render recombinant MVA (rMVA) genetically unstable, leading to the accumulation of mutated rMVA with impaired transgene expression. This represents a major challenge for upscaling and manufacturing of rMVA vaccines. To prevent transgene-mediated negative selection, the continuous avian cell line AGE1.CR pIX (CR pIX) was modified to suppress transgene expression during rMVA generation and amplification. This was achieved by constitutively expressing a tetracycline repressor (TetR) together with a rat-derived shRNA in engineered CR pIX PRO suppressor cells targeting an operator element (tetO) and 3’ untranslated sequence motif on a chimeric poxviral promoter and the transgene mRNA, respectively. This cell line was instrumental in generating two rMVA (isolate CR19) expressing a *Macaca fascicularis* papillomavirus type 3 (MfPV3) E1E2E6E7 artificially-fused polyprotein following recombination-mediated integration of the coding sequences into the DelIII (CR19 M-DelIII) or TK locus (CR19 M-TK), respectively. Characterization of rMVA on parental CR pIX or engineered CR pIX PRO suppressor cells revealed enhanced replication kinetics, higher virus titers and a focus morphology equaling wild-type MVA, when transgene expression was suppressed. Serially passaging both rMVA ten times on parental CR pIX cells and tracking E1E2E6E7 expression by flow cytometry revealed a rapid loss of transgene product after only few passages. PCR analysis and next-generation sequencing demonstrated that rMVA accumulated mutations within the E1E2E6E7 open reading frame (CR19 M-TK) or deletions of the whole transgene cassette (CR19 M-DelIII). In contrast, CR pIX PRO suppressor cells preserved robust transgene expression for up to 10 passages, however, rMVAs were more stable when E1E2E6E7 was integrated into the TK as compared to the DelIII locus. In conclusion, sustained knock-down of transgene expression in CR pIX PRO suppressor cells facilitates the generation, propagation and large-scale manufacturing of rMVA with transgenes hampering viral replication.

## 1 Introduction

Modified Vaccinia virus Ankara (MVA) is a highly attenuated poxvirus strain which has been derived from Chorioallantois Vaccinia virus Ankara (CVA) by over 500 passages on primary chicken embryo fibroblasts (1, 2). Because of its extensive passaging, MVA differs from CVA by the loss of about 30 kb of genomic DNA in 6 major deletion sites (3) and several smaller mutations (4). As a consequence, replication of MVA is restricted to avian cells and very few mammalian cells (5–8). MVA has a proven clinical track record and a high safety profile (9). Therefore, MVA can also be used in immunocompromised animals (10) and humans (11). MVA-BN is an approved prophylactic vaccine against smallpox and monkeypox in the European Union (12) and the USA (13). Moreover, MVA is a versatile vector to deliver vaccine payloads because of its ability to express large and multiple transgenes and the capacity to elicit robust T-cell and antibody responses (14, 15). Since 2020, the recombinant MVA (rMVA) MVA-BN-Filo (16) is approved in combination with Zabdeno as a prophylactic vaccine against Zaire ebolavirus (17). Additionally, multiple MVA based vaccine candidates are under clinical development (18), including more recently candidates targeting e.g. SARS-CoV2 and MERS (19, 20). Apart from prophylactic vaccines, rMVA is also investigated as a potential vector platform for immunotherapeutics (21, 22).

Instability of rMVA, i. e. the tendency to spontaneously loose transgene expression, is a common issue in the field (23–27). While generating rMVA vaccines against different viruses, e.g. Ebola, HIV, SARS-CoV-2, and also immunotherapeutics against HPV- and HERV-induced cancers, we and others commonly found mutated rMVA (mrMVA) that had lost expression of their recombinant antigen after tissue culture passage (23–27). As described by Wyatt et al., although unstable rMVA may be present in virus stocks at concentrations too low for detection unless extensive focus screening or deep-sequencing is performed, they are able to rapidly overgrow the desired rMVA if loss of transgene expression leads to a replication advantage (23). Since production of clinical GMP-grade rMVA for large vaccine seed stocks requires vector expansion, it is important to maintain genetic stability of the transgene over a sufficient number of replication cycles in order to preserve immunogenicity and efficacy of the vaccine. Wang and colleagues showed that rMVA encoding CMV IE1 and pp65 propagated for 10 passages exhibited lower induction of antigen specific T cell responses in preclinical analysis, and detailed characterization of this passaged rMVA revealed frequent loss of the CMV transgenes (26). Even though stability of rMVA is crucial, only few studies systematically investigated the stability of rMVA. Three factors seem to contribute to the instability of some rMVA: the integration locus (23, 27), promotor usage controlling recombinant transgene expression (26), and harmful transgene products (23, 25).

We previously have generated adenoviral vectors as therapeutic vaccine candidates encoding various combinations of optimized *Macaca fascicularis* papillomavirus 3 (MfPV3) and human papillomavirus type 16 early antigens (E1, E2, E6, E7) (28, 29). For a heterologous prime-boost regimen, we aimed towards complementing our vector suite with rMVA [MVA-CR19 (30)] expressing a corresponding set of antigens. Despite our serious efforts we failed to generate these rMVA on the continuous avian AGEI.CR pIX (CR pIX) cell line, regardless of the integration locus and the poxviral promoter usage (31).

Here, we provide evidence that the expression of the papillomavirus early antigens was associated with genetic instability and selection of rMVA mutants escaping transgene expression alongside with replication. Loss of transgene expression was mainly caused by early translation terminations or large deletions depending on the locus of the integrated transgene in the rMVA genome. Suppression of transgene expression in an engineered suppressor cell line (CR pIX PRO) by targeting an operator element on chimeric poxviral promoters via a constitutively expressed tetracycline repressor (TetR) together with a shRNA (Plin-2) targeting a rat perilipin-2 derived 3’ untranslated sequence element (P2TS) on the transgene mRNA restored genetic stability of the rMVA and stabilized transgene expression across several passages. Our data suggest that sustained transgene suppression during generation and amplification of rMVA mitigates the risk for transgene mediated genetic instability and strongly enhances production yields of otherwise hard to produce rMVA.

## 2 Methods

### 2.1 Antigen sequence

Antigen Ii-E1E2E6E7 was generated as previously described (28). The cassette was adapted to human codon usage using the GeneOptimizer Algorithm (Thermo Scientific), excluding canonical vaccinia transcription termination signal T_5_NT (32) and the known instability motifs - runs of G_5_ and C_5_ (23). Approximately 700 bp flanking regions of the DelIII-locus were considered during the optimization run, so that the algorithm not only reduces homology within the antigen but also between antigen and flanking vaccinia sequence.

### 2.2 Cell lines

Adherent AGEI.CR pIX (CR pIX) cells were cultivated in DMEM/F-12 containing 5% fetal calf serum (FCS) as previously described (31). CR pIX cells in suspension were cultivated as previously described (31). Modified AGEI.CR pIX (CR pIX PRO) suppressor cells were generated by transduction of CR pIX cells with a VSV-G pseudotyped lentiviral vector at MOI 1 in the presence of 8 µg/ml polybrene (hexadimethrine bromide, Cat. H9268, Sigma-Aldrich, Germany) coding for the ProVector expression cassette. In detail, the ProVector expression cassette contains Plin2 shRNA under control of a U6 promoter, the tetracyclin repressor gene (tetR) controlled by a CMV promoter, and a puromycin resistance marker gene under the EF1α core promoter. CR pIX PRO cells were cultivated in respective growth medium additionally containing 1 µg/ml puromycin for selection purposes.

### 2.3 Generation of recombinant viruses

Recombinant MVA-CR19 vectors were generated as previously described (33). Briefly, MfPV3 Ii-E1E2E6E7 (28) or GFP was cloned into the shuttle vector SP-CR19III, suitable for integration into MVA’s deletion site III (DelIII) under the control of the MVA E/L-promoter [SSP with one point mutation (33)] or cloned into the shuttle vector pLZAW1, suitable for integration into MVA’s thymidine kinase locus (TK) under the control of the MVA SSP-promoter (34). The shuttle vectors were modified further by inserting two copies of the tetracycline operator sequence (tetO) directly downstream of the MVA promoter and by adding the Plin2 target sequence (P2TS) at the 3’-UTR of the GOI. Recombinant MVA encoding the different genes-of-interest (GOI) were generated by homologous recombination in adherent parental CR pIX or CR pIX PRO suppressor cells that prevent the undesirable expression of the GOI during generation and propagation of the recombinant MVA. To this end, the culture monolayers seeded in a six-well plate were infected with MVA-CR19 with an MOI of 0.05 and transfected with 2 µg of the individual shuttle vector by using the Effectene Transfection Reagent (Qiagen, Hilden, Germany) according to the manufacturer’s instructions. The infected/transfected culture was harvested 2-3 days post-infection/transfection, sonicated, and used for infection of a cell monolayer in a six-well plate format. Resulting foci were validated by PCR and an iterative focus purification procedure was initiated until MVA without the correct GOI expression cassette were absent (usually within 5–8 rounds of focus purification).

For the propagation of focus-purified rMVA, the cell harvest material was sonicated by using a Vial Tweeter (set to 20 s of 100% cycle and 90% amplitude, Hielscher, Germany), and CR pIX PRO cells [grown in suspension at 2×10^6^ cells per ml in 1:1 mixtures of CD-U4 and CD-VP4 media (Merck-Millipore, Darmstadt, Germany)] were inoculated with the individual recombinant MVA vectors at MOI 0.05. Finally, rMVA were harvested 48 h - 72 h post-infection and the TCID50 titer was determined. 3 propagation cycles were needed to generate the viral stocks.

### 2.4 Virus titration

Virus titers were measured by using the tissue-culture-infectious-dose 50 (TCID50) assay. Briefly, 3×10^4^ CR pIX PRO suppressor cells per well were seeded in 96-well plates. Virus-containing material was serially diluted 1:10 in 8 technical replicates and added to the cells. 72 h post-infection the cells were screened for CPE by optical inspection under the microscope. TCID50 was calculated with the Spearman-Karber formula (35).

### 2.5 Antibodies

The following antibodies were used in this study: monoclonal mouse anti-myc antibody (9B11, 1:1000 in western blot, 1:500 in flow cytometry, Cell Signaling Technologies, Danvers, USA), polyclonal rabbit anti-vaccinia (1-VA003-07, 1:5000 in western blot, 1:1000 in flow cytometry and immunostaining, Quartett, Berlin, Germany), monoclonal mouse anti-tubulin (DM1α, 1:1000 in western blot, Santa Cruz Biotechnologies, Heidelberg, Germany), polyclonal goat anti-mouse-HRP (115-036-003, 1:5000, Jackson, West Grove, USA), polyclonal goat anti-rabbit-HRP (P0448, 1:2000 in western blot and immunostaining, Dako, Santa Clara, USA), polyclonal goat anti-mouse-PE (550589, 1:200 in flow cytometry, BD, Franklin Lakes, USA), goat anti-rabbit Alexa Fluor 647 (A21244, 1:200 in flow cytometry, Life Technologies, Eugene, USA).

### 2.6 Western blot

Western blot analysis was performed as previously described (36). Briefly, cells of interest were lysed in TDLB buffer (50 mM Tris, pH 8.0, 150 mM NaCl, 0.1% SDS, 1% Nonidet P-40 and 0.5% sodium deoxycholate) supplemented with protease inhibitors (Complete Mini, Roche, Basel, Switzerland). Total protein concentration of the supernatants was measured by the Bradford method (Protein Assay, BioRad, Feldkirchen, Germany). The proteins were separated on 10% SDS-PAGE under reducing conditions and blotted on a nitrocellulose membrane for western blot analysis. Targets were probed with primary and secondary antibodies as listed above. HRP-labeled secondary antibodies and enhanced chemiluminescence substrate or Femto ECL (Thermo Fisher, Waltham, USA) were used for detection in a Chemilux Pro device (Intas, Göttingen, Germany).

### 2.7 Flow cytometry

Intracellular staining of antigens was performed by using standard methods (36). Cells were fixed and permeabilized with cytofix/cytoperm-Buffer (4% PFA, 1% saponine, in PBS). All washing steps were done with perm/wash-buffer (PBS containing 0.1% saponine). All antibodies were diluted in perm/wash-buffer and incubated for 30 min each. Flow cytometry was performed by using an Attune NxT device (Thermo Fisher, Waltham, USA) with 488 nm and 638 nm excitation and 574/26 nm and 670/14 nm emission filters. Cells were gated on stained, uninfected, and stained mock-MVA-infected cells. Evaluation of data was performed by using Attune NxT software.

### 2.8 Quantification of transgene expression

3x10^4^ CR pIX and CR pIX PRO cells were seeded and infected with the indicated MVA strain at an MOI of 1. After 6 and 24 hpi, cells were harvested into PBS. Total RNA was prepared with RNeasy Mini kit (Cat. 74106, Qiagen, Hilden, Germany) according to manufacturer’s instructions. Impurities from genomic DNA were digested with Turbo DNA-*free* kit (Cat. AM1907, Thermo Fisher, Waltham, USA). Reverse transcription quantitative PCR (RT-qPCR) was performed with Luna Universal Probe One-Step RT-qPCR kit (E3006L, NEB, Ipswich, USA) according to manufacturer’s instructions. 2 µl of total RNA extract were used on a StepOnePlus qPCR cycler (Thermo Fisher, Waltham, USA) with the following protocol: Initial reverse transcription at 55°C for 10 min, followed by initial denaturation at 95°C for 1 min, followed by 40 cycles of denaturation for 15 s at 95°C and annealing/extension for 30 s at 60°C.

Following primer and probes were used: To amplify the transgene, E2 forward primer GATACAGGCTGGGACAAAGTG and E2 reverse primer GATCACTGTTCTGCCGATATGC were used together with the E2 probe Fam-CCTGTACTATGTGCTGCACGGCCT-BHQ1. For normalization on virus infection MVA128L was amplified with MVA128L forward primer CGTTTTGCATCATACCTCCATCTT and the MVA128L reverse primer GCGGGTGCTGGAGTGCTT, together with the MVA128L probe Fam-AGGCATAAACGATTGCTGCTGTTCCTCTGT-BHQ1.

For relative quantification of the transgene expression, Ct values were normalized according the following equation (37), using the primer efficiency calculated by the StepOnePlus software:


fold reduction of transgene expression=1effE2    CtCR pIX−CtCR pIX PROeffMVA128L    CtCR pIX−CtCR pIX PRO

### 2.9 Immunostaining of foci

Immunostaining was used to visualize foci, as described previously (38). 3×10^5^ CR pIX or CR pIX PRO cells per well were seeded in 24-well plates. Cells were infected with an MOI of 0.01 in 1 ml DMEM/F-12 without additives. 2 h post-infection, medium was exchanged to DMEM/F-12 with 5% FCS. 48 h post-infection, cells were fixed with ice-cold acetone/methanol solution (1:1, v/v) and blocked with blocking buffer (PBS containing 3% BSA) overnight at 4°C. Cells were sequentially stained by using an anti-vaccinia antibody and anti-rabbit-HRP, both incubated for 1 h at RT with gentle agitation. All wash steps and antibody dilution steps were conducted in blocking buffer. Finally, foci were stained with TrueBlue Peroxidase Substrate (5510-0030, Seracare, Milford, USA) until foci were visible (usually 5-10 min); subsequently the reaction was stopped with water. Pictures were taken with an inverted microscope (BZ-9000, Keyence, Frankfurt, Germany).

### 2.10 Multistep growth curve

To analyze virus replication, a multi-step growth curve was conducted as described previously (5). 3×10^5^ CR pIX and CR pIX PRO cells per well were seeded in 24-well plates. Cells were infected at an MOI of 0.05 in 200 µl DMEM/F-12 without additives. 45 min post-infection, cells were carefully washed once with PBS and incubated with a medium containing 5% FCS. Cells and supernatant were harvested at 0, 24, 48, and 72 h after adsorption, freeze-thawed thrice, sonicated for 1 min, and titrated as mentioned above.

### 2.11 Passaging of rMVA

For analysis of genetic stability of rMVA, the viruses were passaged on parental CR pIX and CR pIX PRO suppressor cells, similar to the protocol of Kremer and colleagues (38). 1.2×10^6^ cells per well were seeded in a 6-well plate 24 h before infection. Cells were infected at an MOI of 0.05 in 1 ml DMEM/F-12 without additives. 2 h post-infection, medium was exchanged to DMEM/F-12 with 5% FCS. 48 h post-infection, cells and supernatant were harvested (=passage 1), freeze-thawed three times, and additionally sonicated three times for 1 min in a cup sonifier. New cells were again infected with 1 ml of a 1:1000 dilution of the virus material from the previous passage. This was repeated to a maximum number of 10 passages. All obtained virus material was stored at -80°C.

To obtain single foci, cell monolayers were infected with different dilutions and covered with growth medium containing 0.8% low melting agarose. 72 h post-infection, foci were picked in 300 µl DMEM/F-12, freeze-thawed three times, sonicated for 1 min, and amplified by infection of 3×10^5^ cells per well in 24-well plates.

### 2.12 Genotyping of MVA

For genotyping of transgene insertion loci, PCR was utilized. Genomic DNA of either focus-purified or bulk material of virus-infected cells or virus stocks was prepared with a Quick-DNA MiniPrep kit (D3025, Zymo, Freiburg, Germany) according to the manufacturer’s instructions. PCR was conducted with Q5 High-fidelity DNA polymerase (M0491L, NEB, Ipswich, USA) according to standard protocols. Primers were taken from literature (33, 38) and are listed in **Table 1**. Analysis was done by electrophoresis in 0.5%, 0.8% or 1.5% agarose TBE gels, depending on the amplicon size.

**Table 1:** Primers for genotyping of MVA.

Primer	Primer sequence	Expected size	T_M_ and elongation time
DelIII f x r	GATGAGTGTAGATGCTGTTATTTTG x GCAGCTAAAAGAATAATGGAATTG	Wildtype: 446 bp With transgene: 5143 bp	61°C 3.5 min
TK f x r	CTCTCTAGCTACCACCGCAA x CACTACGGTGGCACCATCTAA	Wildtype: 920 bp With transgene: 5094 bp	67°C 4.33 min
DelVI f x r	CGTCATCGATAACTGTAGTCTTG x TACCCTTCGAATAAATAAAGACG	702 bp	60°C 0.5 min
DelIII wide III3-f x III2-r	GCCGGTATTGGCATACAGTTC x GCAATTGTCAGTTAACACAAGTCC	Wildtype: 9471 bp With transgene: 14169 bp	65°C 11.5 min

For Sanger sequencing analysis, PCR amplicons of bulk material were purified from agarose gel by using QIAquick Gel Extraction Kit (28706X4, QIAGEN, Hilden, Germany), ligated into pJET1.2/blunt (K1231, Thermo Fisher Scientific, Waltham, USA) and used for transformation of E. coli. Plasmids were isolated from single clones and sequenced.

### 2.13 Next-generation sequencing

Exogenous cellular gDNA was depleted from virus-containing material as described previously (30). Briefly, virus stock or virus-containing material was precipitated by the addition of polyethylene glycol to a final concentration of 8% (w/w), incubated for 30 min on ice, and subsequently centrifuged at 6600 g for 1 h. The translucent pellet was dissolved in PBS and exogenous gDNA was digested with 8 units of TurboDNAse for 1 h, followed by adding 20 mM EDTA and heat-inactivation at 80°C for 10 min. Viral gDNA was prepared with a Quick-DNA MiniPrep kit (D3025, Zymo, Freiburg, Germany) according to the manufacturer’s instructions. 10 µl of each gDNA were barcoded for NGS with the Nextera XT DNA library Prep kit (FC-131-1096, Illumina, San Diego, USA) according to the manufacturer’s instructions. NGS was performed with the NextSeq500 system by using a NextSeq500/550 High Output Kit v2.5 with 300 cycles (20024905, Illumina, San Diego, USA). FastQ files were evaluated with CLC Genomics Workbench 22 (Qiagen, Hilden, Germany). Obtained sequences were assembled by using derivatives of the MVA-CR19 genome sequence (GenBank accession number KY633487.1) with Ii-E1E2E6E7 transgene integrated into DelIII or TK locus. Normalized transgene coverage was calculated with the formula:


$$\%=\frac{\frac{read\;coverage\;transgene\;of\;passage\;x}{read\;coverage\;of\;MVA056L\;of\;passage\;x}}{\frac{read\;coverage\;transgene\;of\;seed\;stock}{read\;coverage\;of\;MVA056L\;of\;seed\;stock}}$$

## 3 Results

### 3.1 Tet repressor- and shRNA-mediated knock-down of MVA transgene expression

We have demonstrated previously that a fusion protein comprising the early antigens E1, E2, E6 and E7 of *Macaca fascicularis* papillomavirus type 3 (MfPV3) are well suited to induce potent T cell responses in outbred CD1 and OF1 mice when delivered via DNA or adenoviral vectors (28). Repeated attempts to generate a recombinant MVA (rMVA-CR19) expressing the corresponding Ii-E1E2E6E7 fusion protein for booster immunization purposes was hampered by the stepwise loss of transgene positive foci alongside the focus selection process. This led us to hypothesize that expression of the transgene Ii-E1E2E6E7 imposed a disadvantage on rMVA propagation.

**Figure 1:**
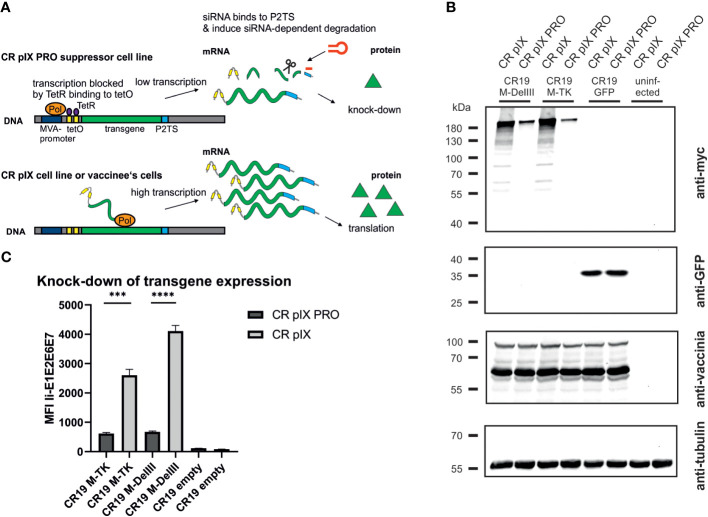
Transgene expression of CR19 M-TK and CR19 M-DelIII on parental CR pIX and CR pIX PRO suppressor cell line. **(A)** Schematic overview of TetR/tetO and Plin2 shRNA/P2TS mediated transgene suppression. Colors and shapes resemble the following: orange: MVA RNA polymerase; dark blue: MVA promoter; yellow: tetO; violet: TetR; light blue: P2TS; green: transgene DNA/mRNA; red hairpin: Plin2 shRNA; green triangle: transgene product **(B)** Western blot analysis of CR pIX PRO suppressor cells and CR pIX cells infected with rMVA coding for MfPV3 Ii-E1E2E6E7 (integrated into DelIII or TK locus) controlled by tetO and P2TS, or infected with rMVA coding for GFP lacking tetO and PT2S. Cells were infected at an MOI of 0.5 and harvested 48 hours post infection (hpi). Antibodies used are indicated on the right. **(C)** Expression analysis of CR pIX PRO and CR pIX cells infected with rMVA coding for MfPV3 Ii-E1E2E6E7 controlled by tetO and P2TS. Cells were infected at an MOI of 0.1 and harvested 48 hpi. Cells were stained with rabbit anti-vaccinia and goat anti-rabbit AF647 for MVA infection and mouse anti-myc and goat anti-mouse PE for transgene expression. Cells were gated on vaccinia-positive cells using the fluorescence background of cells infected with MVA without any antigen. MFI of PE out of AF647 positive cells is represented as mean with SEM, Statistical analysis was done with an unpaired t-test. ***p< 0.0005; ****p<0.00005; n=3 biological replicates.

Hence, we herein aimed to develop a system to suppress transgene expression during the generation, selection and amplification of the rMVA. For this purpose, we first modified the AGEI.CR pIX cell line (CR pIX) by means of lentiviral transduction to constitutively express the tetracycline repressor (TetR) together with a rat perilipin-2 (Plin2) shRNA yielding AGEI.CR pIX PRO (CR pIX PRO). Complementary, two tetracycline operator sequences (tetO) and a Plin2 shRNA target sequence (P2TS) were integrated into the 5’ and the 3’ UTR of the transgene expression module of the transfer plasmid used to generate rMVA via *in vitro* recombination (IVR). According to these design features, residual mRNAs possibly resulting from incomplete TetR/tetO-mediated transcription repression will be degraded via binding of Plin2 shRNA to the 3’ PT2S on the transcript, collectively resulting in a sustained knock-down of toxic transgene expression. The CR pIX PRO suppressor cell line used in conjunction with the tetO/Plin2 transfer plasmids for IVR, rMVA focus selection and subsequent rMVA amplification is in the following referred to as ProVector system (**Figure 1A**).

Performing the IVR with the transfer plasmids encoding the presumably toxic MfPV3 Ii-E1E2E6E7 fusion protein, now flanked by the tetO/P2TS control elements, in the CR pIX PRO suppressor cells and applying the ProVector system for subsequent rMVA selection, we eventually succeeded to generate two rMVA: MVA-CR19-TK (short CR19 M-TK) and MVA-CR19-DelIII (CR19 M-DelIII) with the MfPV3 Ii-E1E2E6E7 transgene sequence integrated either into the thymidine kinase (TK) locus or, alternatively, in the deletion III (DelIII) locus, respectively (**Supplementary Figure 1**). Virus stocks for the subsequent experiments were produced starting from single foci via 3 rounds of expansion on CR pIX PRO suppressor cells.

Specific knock-down of Ii-E1E2E6E7 expression by CR pIX PRO suppressor cells was initially proven via western blot analysis and quantified by flow cytometry using a C-terminally fused myc-tag for expression monitoring of the fusion protein, due to unavailability of MfPV3 early protein-specific antibodies and HPV-antibodies are not cross-reactive (**Figures 1B, C**). Expression of Ii-E1E2E6E7 was clearly reduced in CR pIX PRO suppressor cells as compared to parental CR pIX cells, when infected with CR19 M-TK and CR19 M-DelIII, respectively, suggesting successful suppression of transgene expression (**Figure 1B**). GFP expression was comparable in parental CR pIX cells and CR pIX PRO suppressor cells following infection with rMVA-GFP lacking tetO and P2TS, respectively. This demonstrates that the presence of the TetR and Plin2 shRNAs do not impact transgene expression per se. Flow cytometry analysis confirmed approximately 4.3-fold and 6-fold suppression of Ii-E1E2E6E7 expression in CR pIX PRO suppressor cells compared with the parental CR pIX cells 48 hpi with CR19 M-TK and CR19 M-DelIII (**Figure 1C**; **Supplementary Figure 2**). Ii-E1E2E6E7 mRNA levels were determined by RT-qPCR and shown to be reduced in CR pIX PRO suppressor cells by a factor of 5.8 (CR19 M-DelIII) or 1.98 (CR19 M-TK) at 6 hpi, respectively, and by a factor of 11.43 (CR19 M-DelIII) or 2.96 (CR19 M-TK) after 24 h (**Supplementary Figure 3**). The observed reduction of Ii-E1E2E6E7 transcripts level in the CR pIX PRO suppressor cells might be underestimated, and mainly capture the impact of TetR-mediated suppression of transcription, as exact quantification of siRNA-mediated mRNA degradation is hard to achieve by means of RT-qPCR (39).

### 3.2 rMVA expressing MfPV3 Ii-E1E2E6E7 has a replication disadvantage on parental CR pIX cells which can be rescued on CR pIX PRO suppressor cells

**Figure 2:**
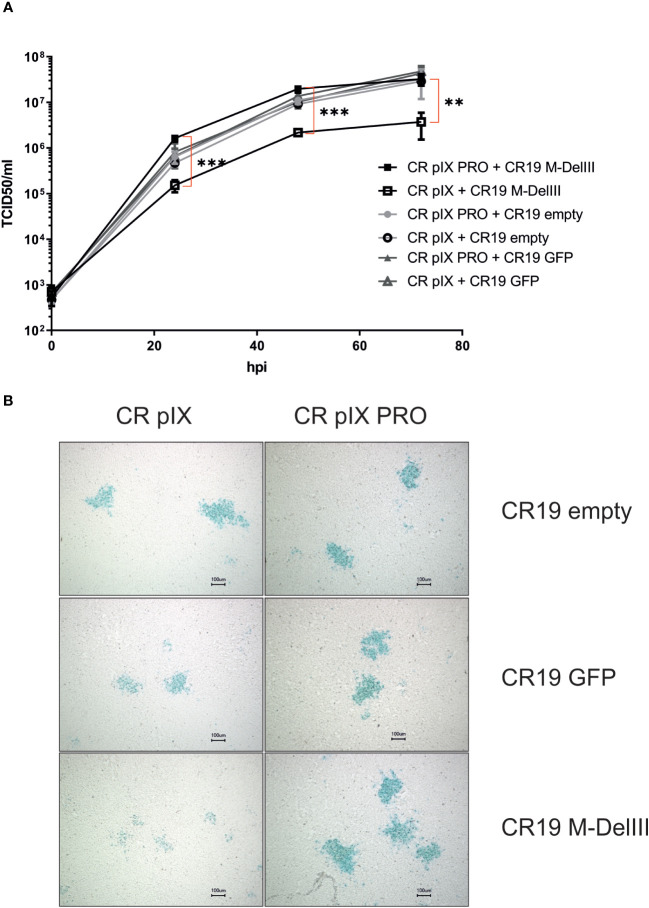
Replication kinetics and focus morphology of CR19 empty, CR19 GFP and CR19 M-DelIII on parental CR pIX and CR pIX PRO suppressor cell line. **(A)** Multiple growth step curves for the indicated CR19 variants on CR pIX and CR pIX PRO cells. Cells were infected at an MOI of 0.05. Samples taken at 0, 24, 48, and 72 hpi were titrated on CR pIX PRO suppressor cells using the TCID50 method. Statistical analysis was done with an unpaired t-test. Data points represent mean with SEM, **p< 0.005; ***p<0.0005 n=3 independent biological replicates. **(B)** CR pIX and CR pIX PRO suppressor cells were infected at an MOI of 0.01 with the indicated rMVA-CR19. 48 hpi, cells were fixed and stained with an anti-vaccinia antibody, an HRP-coupled secondary antibody and KPL Trublue substrate. Photos were taken on a Keyence inverted microscope at a magnification of 10. Bar represents 100 µm. Shown foci are representative for the respective conditions.

Following successful generation of an rMVA-MfPV3-Ii-E1E2E6E7 virus stock on CR pIX PRO suppressor cells, we set out to test the hypothesis that (i) expression of certain potentially harmful transgenes can have a detrimental impact on virus replication and that (ii) replicative capacity can be restored in such cases by restricting expression of such transgenes. For this purpose, replication kinetics of CR19 M-DelIII and, for comparison, CR19 GFP, and CR19 empty were measured under restricting and non-restricting conditions. CR pIX PRO suppressor cells and non-modified parental CR pIX cells were infected with the respective rMVA at an MOI of 0.05, and a multistep growth curve was generated by harvesting infected cells together with cell supernatant after 0, 24, 48, and 72 hpi. Titration on CR pIX PRO suppressor cells showed that the titer of CR19 M-DelIII was significantly reduced by a factor of approximately 10 when propagated on CR pIX cells compared with CR pIX PRO suppressor cells at all time points (**Figure 2A**). As a reference, virus titer of CR19 M-DelIII on CR pIX PRO cells was equal to the titers of both control viruses CR19 empty and CR19 GFP that in turn displayed no differences on both cell lines. Consistent results were obtained when comparing the focus size of the rMVA at 48 hpi (MOI of 0.01) (**Figure 2B**; **Supplementary Figure 4**). Immunostaining with a vaccinia-specific antibody revealed comparable foci sizes formed by CR19 empty, CR19 GFP, and CR19 M-DelIII on CR pIX PRO suppressor cells. In contrast, smaller sized foci with less intense staining were notified following infection of the parental CR pIX cells CR19 M-DelIII expressing the Ii-E1E2E6E7 transgene, but not for CR19 GFP and CR19 empty.

Taken together, this suggests that unrestricted expression of the MfPV3 Ii-E1E2E6E7 antigen results in a replication disadvantage for CR19 M-DelIII and, consequently, reduced virus titers and mitigated virus spread. This replication deficit can be restored when Ii-E1E2E6E7 expression is knocked-down in CR pIX PRO suppressor cells.

### 3.3 Replication of CR19 M-DelIII, but not of CR19 M-TK, is restored upon passaging on parental CR pIX cells

Provided the MfPV3-Ii-E1E2E67 transgene has negative impact on the MVA replicative capacity under non-restricting conditions, the transgene might also have an influence on the genetic stability of the rMVA by imposing a strong negative selection on such transgene-expressing rMVA. To test this, we first generated a CR19 M-DelIII and CR19 M-TK seed virus stock (defined as passage 0) by expanding the originally selected, positive foci (i.e. passage -3) in three consecutive amplification steps on CR pIX PRO suppressor cells, respectively. Such seed virus stocks were then serially passaged side-by-side on the parental CR pIX cells and on CR pIX PRO suppressor cells for 10 passages as described by Kremer et al. (38). Harvested virus samples were subsequently titrated under restricting conditions on CR pIX PRO suppressor cells (**Supplementary Figure 5**).

**Figure 3:**
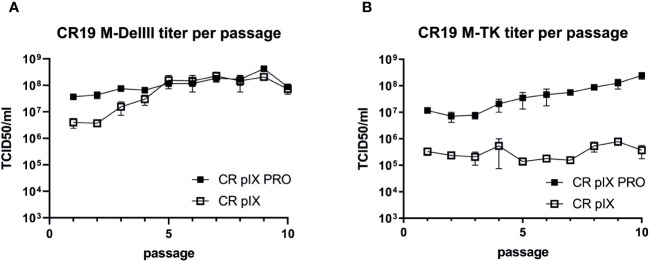
Growth kinetic (titer per passage) of CR19 M-DelIII **(A)** and CR19 M-TK **(B)** passaged on either parental CR pIX or CR pIX PRO suppressor cells. Samples of each passage were titrated on CR pIX PRO suppressor cells by using the TCID50 method. TCID50s for each passage are indicated. Data points represent the mean with SEM, n=2 independent biological replicates.

Titration of CR19 M-DelIII virus samples harvested after each passage on CR pIX PRO suppressor cells yielded similar titers across all passages with a trend towards marginally higher titers in later passages, which did, however, not reach statistical significance (**Figure 3A**). Starting from the same seed virus stock (passage 0), CR19 M-DelIII lost already after 1 passage on the parental CR pIX cells one order of magnitude in virus titer as compared to the same virus passaged on CR pIX PRO suppressor cells (**Figure 2A**). This observation was in line with previous findings suggesting a replication disadvantage of CR19 M-DelIII under non-restricting conditions in the parental CR pIX cells (**Figure 2**; **Supplementary Figure 4**). Beginning with passage 3, the titers of CR pIX-passaged CR19 M-DelIII increased and reached the same level as CR-pIX-PRO-passaged CR19 M-DelIII from passage 5 onwards.

A different effect was observed when CR19 M-TK was passaged on the two cell lines: Whilst CR19 M-TK, similar to CR19 M-DelIII, experienced a significant loss in titer after a single passage on the parental CR pIX cells, the titers of CR19 M-TK did - unlike notified for CR19 M-DelIII - not increase while being passaged on the parental CR pIX cells and consistently remained approximately 100-fold below the levels obtained for CR19 M-TK passaged on CR pIX PRO suppressor cells (**Figure 3B**). In general, CR19 M-TK exhibited slightly lower virus titers as compared with CR19 M-DelIII across all passages on both cell lines.

### 3.4 Passaging on CR pIX cells leads to rapid loss of Ii-E1E2E6E7 expression

**Figure 4:**
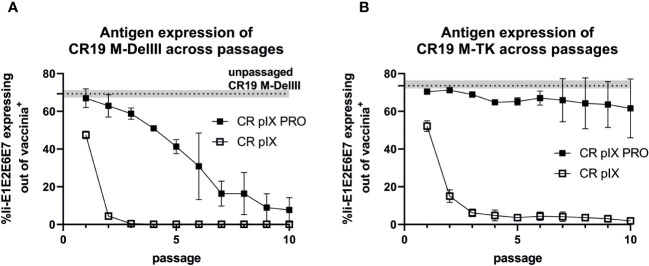
Expression analysis of CR19 M-DelIII **(A)** and CR19 M-TK **(B)** passaged on either parental CR pIX or CR pIX PRO suppressor cells by using flow cytometry. CR pIX cells were infected at an MOI of 0.1 (CR19 M-DelIII) or MOI of 0.01 (CR19 M-TK) with virus samples obtained after each passage and were harvested 48 hpi. Cells were stained with rabbit anti-vaccinia and goat anti-rabbit AF647 for MVA infection and mouse anti-myc and goat anti-mouse PE for transgene expression. Cells were gated on vaccinia-positive cells using cells infected with MVA without any antigen. %gated Ii-E1E2E6E7 expressing cells out of vaccinia antigen positive cells represents myc-positive cells out of vaccinia-positive cells. Dashed line represents Ii-E1E2E6E7-expressing out of vac^+^ cells of passage 0 of CR19 M-DelIII or CR19 M-TK used as starting material for passaging on both cell lines. Data points represent mean with SEM, n=2 independent biological replicates.

We next quantified Ii-E1E2E6E7 transgene expression under non-restricting conditions in parental CR pIX cells of CR19 M-DelIII samples harvested after each passage on either CR pIX or CR pIX PRO suppressor cells, respectively, in a high-throughput flow-cytometry-based assay. Infected CR pIX cells were co-stained to monitor intracellular expression of Ii-E1E2E6E7 (via its myc-tag) and vaccinia proteins (polyclonal anti-vaccinia-antibody) 48 hpi (**Figure 4**). As controls, uninfected cells and CR19-empty-infected cells were used to gate for CR19-infected cells (vac^+^), and CR19 M-DelIII to gate for Ii-E1E2E6E7 expression out of vac^+^ cells (**Supplementary Figure 6**). A rapid reduction in the frequency of Ii-E1E2E6E7 expressing cells among the CR19 M-DelIII-infected cells was observed. This was already apparent with virus samples derived from passage 1 on CR pIX cells (**Figure 4A**). After only 3 passages on the parental CR pIX cells, expression of Ii-E1E2E6E7 was lost. In contrast, expression of Ii-E1E2E6E7 could be observed with CR19 M-DelIII passaged on CR pIX PRO suppressor cells throughout all 10 passages. However, even suppression of transgene expression in the ProVector system could not prevent a continuously decreasing fraction of Ii-E1E2E6E7 expressing cells amongst vaccinia virus positive cells with passages from 70% to only 10%. A rapid reduction in the frequency of Ii-E1E2E6E7^+^ cells amongst vaccinia virus antigen positive cells was also observed for CR19 M-TK passaged on CR pIX cells (**Figure 4B**). Interestingly, no significant difference in the fraction of Ii-E1E2E6E7 expressing cells was observed with passage level of CR19 M-TK grown on CR pIX PRO suppressor cells.

In conclusion, this experiment suggests a strong negative selection on rMVA expressing Ii-E1E2E6E7. Suppression of transgene expression by CR19 pIX PRO suppressor cells led to prolonged production of Ii-E1E2E6E7. Remarkably, the integration site had a major impact on the stability of transgene expression: Whereas expression of the DelIII-inserted transgene continuously declined upon passaging, even when the virus was passaged under restricting conditions, the TK-locus-inserted transgene appeared to be maintained more stably, but was strictly dependent on passaging on CR19 pIX PRO suppressor cells.

### 3.5 Loss of Ii-E1E2E6E7 expression from the DelIII locus is mainly caused by transgene deletions (CR19-M-DelIII)

We next investigated whether the reduction of Ii-E1E2E6E7-expressing CR19-M-DelIII- and CR19-M-TK-infected cells is caused by alterations in the DNA sequence of the transgene expression cassette. PCR analyses on single foci from selected passages were performed with primers that bind in the flanking regions of the DelIII and TK locus, respectively (38). In line with the transgene expression analysis above, the fraction of correctly sized PCR amplicons for CR19 M-DelIII quickly declined with the number of passages on CR pIX cells (**Supplementary Table 1**). Some foci, which proved positive with vaccinia-specific primers, showed no specific DelIII amplicon, neither the full length, not a shortened PCR product. This may be explained by acquisition of mutations or deletions in the primer binding sites. In contrast, the expected PCR amplicon could be detected in almost all foci of CR19 M-DelIII passaged on CR pIX PRO.

**Figure 5:**
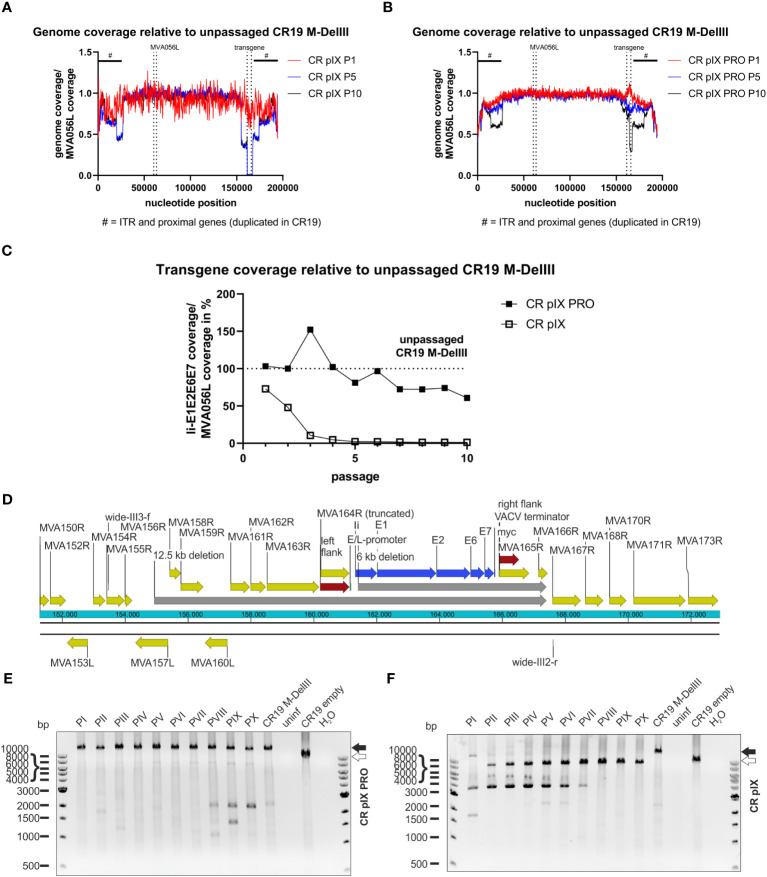
Deletions in the context of the DelIII integration locus across passaging. NGS read coverage across the whole recombinant viral genome from passages P1, P5, and P10, generated on **(A)** parental CR pIX or **(B)** CR pIX PRO suppressor cells. Genome coverage of the respective passages was normalized to genome coverage of the unpassaged rMVA, normalized to MVA056L, respectively. The position of the transgene, and of MVA056L used for normalization, is indicated by vertical dashed lines, the position of ITRs and proximally duplicated genes is indicated by a black scale bar marked with #. **(C)** Depicted is the quotient of the mean read coverage of the entire Ii-E1E2E6E7 transgene integrated into DelIII and the mean read coverage of the essential MVA056L gene (MVA DNA polymerase, E9L) of CR19 M-DelIII passaged on parental CR pIX cells or CR pIX PRO suppressor cells, normalized to the unpassaged rMVA. Dashed line represents normalized read coverage of unpassaged rMVA used as starting material for passaging on both cell lines. **(D)** Schematic overview of DelIII integration locus. Yellow marks MVA genes; red marks homologous sequence flanks used for integration into DelIII locus; blue marks the transgene; grey marks potential deletions postulated by NGS. Genotyping of rMVA passaged on CR pIX PRO suppressor cells **(E)** and parental CR pIX cells **(F)** cells by agarose gel analysis of PCR products obtained with the primer pair wide-III3-f and wide-III2-r. Expected size of CR19 M-DelIII: 14169 bp (black arrow); expected size of CR19 empty: 9471 bp (white arrow).

However, this analysis does not exclude short deletions, point mutations or insertions/deletions of few bases that might result in frame shifts or truncated products. Thus, next-generation sequencing (NGS) was employed as an unbiased method to deeply characterize the genetic integrity of the passaged rMVA. Predominantly viral DNA was isolated from CR-pIX- and CR-pIX-PRO-passaged CR19 M-DelIII and CR19 M-TK by depleting cellular gDNA, and subjected to Illumina NextSeq500 deep-sequencing. The resulting reads were aligned to the CR19 M-DelIII guiding sequence (**Supplementary Table 2**). The obtained coverage maps revealed a wide deletion at the DelIII locus when CR19 M-DelIII had been passaged on parental CR pIX cells (**Figures 5A, B**; **Supplementary Figure 7A**), whereas the central region of the viral genome was stable. This shows that recombination especially affects the transgene (zero coverage in P5 and P10) and adjacent regions (normalized coverage below 0.5). To quantify the fraction of the DelIII locus deletion along the passages, the mean read coverage of the transgene Ii-E1E2E6E7 was normalized to the mean read coverage of the essential MVA-DNA polymerase gene locus (MVA056L) (**Figure 5C**; **Supplementary Table 2**). The normalized Ii-E1E2E6E7 mean read coverage of CR19 M-DelIII rapidly dropped to almost 0% within three passages on CR pIX cells, whereas normalized mean read coverage of Ii-E1E2E6E7 of CR–pIX-PRO-passaged CR19 M-DelIII was overall high with a trend towards some decline at later passages.

Aligning the NGS reads of CR pIX-passaged CR19 M-DelIII with the expected CR19 M-DelIII genome sequence also showed a reduction in read coverage upstream of the transgene (**Supplementary Figure 7A**), spanning from MVA157L to MVA164R, and also a complete absence of MVA165R and parts of MVA166R, both downstream of the transgene. Thus, this comprises not only the deletion of the transgene but also large sequence parts flanking the DelIII locus and extending beyond the flanking sites used for homologous recombination during generation of the recombinant virus (**Figure 5D**).

CR-pIX-PRO-passaged CR19 M-DelIII exhibited a slight reduction in read coverage in passages 5-10. Detailed analysis revealed reduced read coverage spanning from the middle of the E2 gene within the transgene until MVA165R (nucleotide position 161317 to 165771, **Figure 5B**; **Supplementary Figures 7B, C**), indicative for a truncation of the transgene. This may explain the continuously decreasing fraction of Ii-E1E2E6E7 expressing cells amongst vaccinia virus positive cells from 70% (passage 1) to only 10% (passage 10).

To confirm the deletions found by NGS, a PCR analysis with a primer pair spanning from MVA155R to MVA167R was done with the same DNA preparation used for NGS (**Figures 5E, F**). This PCR analysis confirmed fast deletion of the transgene from the DelIII locus when passaged on parental CR pIX cells (**Figure 5F**), resulting in bands of a lower molecular weight than the expected full-length PCR amplicon of 14169 bp (CR19 M-DelIII; black arrow, **Figure 5F**). Moreover, frequent detection of bands of a lower molecular weight than the expected empty DelIII integration site were observed (< 9471 bp, white arrow). These bands resemble deletions not only of the transgene itself but also of neighboring genes upstream and downstream of the DelIII locus. This PCR analysis was repeated for an independent replicate of the passaging experiment (without depletion of cellular gDNA) with similar results (**Supplementary Figure 8**).

### 3.6 Early translational stop as a result of mutations impair transgene expression from the TK locus under non-restricting conditions (CR19 M-TK)

**Figure 6:**
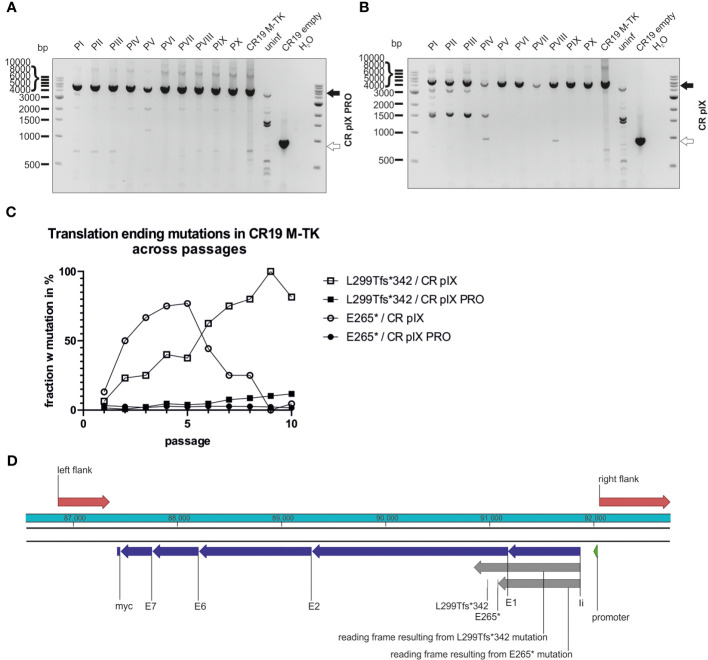
Mutations leading to truncated proteins in the context of TK integration locus across passages. Genotyping of rMVA generated by passaging on CR pIX PRO suppressor **(A)** and parental CR pIX **(B)** cells by using PCR and primer pair TK f and TK r. Expected size of CR19 M-TK of 5094 bp (black arrow) and expected size of CR19 empty of 920 bp (white arrow). **(C)** The fraction of rMVA mutants among CR19 M-TK carrying the early translational stop mutations L299Tfs*342 or E265* across the passages on either CR pIX or CR pIX PRO cells is plotted against the passage number. **(D)** Schematic overview of the transgene in the TK locus. Red marks homologous sequences used for integration into TK locus; blue marks the transgene’s subunits; grey marks reading frames resulting from mutations that lead to early translational stops.

In contrast to CR19 M-DelIII passaged on CR pIX, CR19 M-TK bands resembling shortened PCR products as a result of deletions represented only a minor fraction in the PCR analysis (**Figures 6A, B**; **Supplementary Table 1**). This PCR analysis was repeated for an independent replicate of the passaging experiment and the results were similar (**Supplementary Figure 9**). This was also confirmed by the deep-sequencing analysis of the passaged CR19 M-TK that revealed no difference in normalized transgene coverage between the two cell lines (not shown). However, instead of deletions, specific mutations within the transgene could be observed (**Figures 6C, D**). Passaging of CR19 M-TK on parental CR pIX cells led to the early accumulation of a virus variant harboring a specific guanine to thymidine exchange resulting in the transition of E265 (GAG) to an early stop codon (TAG; referred to as E265*). The fraction of the E265* rMVA mutant among all rMVA peaked at passage 5, but then declined again until passage 9. Furthermore, during passaging on CR pIX, a variant carrying an insertion of guanine after nucleotide position 893 emerged. This insertion results in a frameshift beginning at L299 leading to an early translational stop at position 342 and thus to a truncated protein, referred to as L299Tfs*342. L299Tfs*342 could already be detected in about 5% of the recovered reads of passage 1 on CR pIX cells and accumulated until all recovered reads in passage 9 exhibited this mutation. Both mutations found by deep-sequencing could be verified by Sanger sequencing of pJET1.2/blunt-subcloned PCR amplicons of passage 3 and 10 (**Supplementary Figure 10**). L299Tfs*342 could also be observed among CR19 M-TK passaged on CR pIX PRO cells, albeit only in a very small fraction of the recovered reads. The frequency of this mutant increased only gradually so that at passage 10 the fraction of reads without the L299Tfs*342 was still approximately 90%.

## 4 Discussion

Several recombinant viral vectors have been licensed as vaccines since 2010, among them, in 2020, the recombinant MVA-based Ebolavirus vaccine Mvabea carrying filovirus antigens (40). These recent advancements underscore the potential of viral vector vaccines. Conventionally recombinant MVA are generated by means of homologous recombination between the poxviral genome and a shuttle vector (41). However, depending on the design of the antigen expression cassette, generation may be difficult or even unsuccessful, as we have experienced for an artificial papillomavirus antigen, MfPV3 Ii-E1E2E6E7. Others have reported similar observations (23–27, 42, 43) but only few comparative studies were conducted to analyze this systematically (23, 26, 27). Even in cases when generation of recombinant MVA is successful, their propagation and the generation of bulk material for larger preclinical studies or GMP-grade drug substance or drug product for clinical trials may suffer from transgene instability leading to the emergence of mutated recombinant MVA (mrMVA) with reduced or abrogated antigen expression or modification of the transgene product.

To address this limitation we have implemented a system that conditionally represses MVA-driven transgene expression in an engineered production cell line (CR pIX PRO) and thus supports the generation and expansion of rMVA encoding otherwise difficult to express transgene products. Remarkably, the tetracycline repressor (TetR) in combination with a functional shRNA, both provided from the engineered CR pIX PRO suppressor cell line, was capable to clearly reduce the MVA-mediated transgene expression. In the specific case of the MfPV3-derived Ii-E1E2E6E7 antigen this system eventually enabled the generation of the rMVA, that had failed using unmodified CR pIX cells in earlier attempts.

When e.g. CR19 M-DelII was propagated in non-restricting CR pIX cells, it replicated to lower titers, foci were phenotypically smaller, and more than 90% of the virus had lost antigen expression within three passages. Detailed sequence analysis showed that concomitantly with the loss of antigen expression, mrMVA variants emerged that quickly outcompeted the original rMVA within only few passages. One explanation is that expression of the foreign antigen may be harmful to the host cell. Impaired replication leads to fewer progeny of rMVA expressing the desired antigen compared to mrMVA. Antigen expression thus exerts a negative selection pressure leading to reduced viral fitness in a classical Darwinian “survival of the fittest” competition. Although we cannot completely exclude that cell-type specific factors might impact stability, there is good reason to assume that generation or propagation of rMVAs expressing our MfPV3 Ii-E1E2E6E7 on primary CEF, DF1 or other cell lines would have resulted in comparable instability issues (23, 26, 27).

The loss of rMVA within few rounds of passaging for two different rMVA (TK- and DelIII-integration) carrying the same transgene confirms the general conclusion of negative selection within the virus population. Side-by-side comparison of viruses led to the interesting observation that the mode of antigen suppression can follow different pathways. In the case of DelIII integration site, the transgene is removed by deletions that range from partial transgene deletion to complete deletion even extending into flanking non-essential viral genes, similar to results reported by Wyatt et al. (23). In contrast, in the case of TK insertion, no deletions but rather mutations within the transgene occurred. Here, we observed either a premature stop codon or insertion of a single base causing a frameshift leading to a stop codon in the shifted frame. As a result, a considerably shortened protein variant was expressed that presumably is less toxic to the infected cell. It is plausible that the presence of an essential viral gene next to the TK locus, i.e. MVA085R (Copenhagen J1R) (44), hinders the emergence of viable deletions mutants, thus leading to a genetically more stable region within the genome (45). Interestingly, this leads to the emergence of variants avoiding transgene expression by subtle point mutations, leading to truncated Ii-E1E2E6E7 antigen variants, which seems not to mitigate the fitness loss to the degree achieved by full deletion.

The observed replication capacity of the CR19 M-DelIII-virus population reached wild-type levels once the population was dominated by mrMVA, but this was not the case for the CR19 M-TK-virus population. In line with this, the initially prevalent CR19 M-TK mutant virus, E265*, was subsequently replaced by the L299Tfs*342 variant that (by inference) seems to have a fitness advantage over E265*. The slightly quicker appearance of mrMVA in the case of DelIII-integrations suggests that poxviruses tend to preferably acquire deletions over specific mutations, though this remains speculative given the low number of studied examples. Nevertheless, these observations argue for rather selecting the TK locus over DelIII as more stable integration site when generating rMVA or even more stable integration sites e.g. IGR3 or between I8R and G1L (23, 46).

The TetR/tetO system has been described earlier to conditionally regulate transgene expression in adenoviral vectors (47), and was shown to enhance the genetic stability (48). The concerted action of a production cell line expressing the TetR/Plin2 shRNA (CR pIX PRO suppressor cells) and the transgene expression cassette flanked by a 5’ tetO and a 3’ shRNA target sequence (P2TS) not only augmented generation of the rMVA, but also led to a considerable increase in stability upon propagation and expansion of the virus.

In the repressing CR pIX PRO suppressor cell line, higher virus titers, larger foci, and prolonged retention of the transgene upon passaging were observed. However, suppression was not complete and mrMVA also arose during the passaging experiment, though with a certain delay and, if at all, at much lower frequencies, especially for CR19 M-TK. More than 90% correct virus even after 10 passages constitutes a promising potency for use of such an rMVA as a vaccine, depending on the number of passages that are required in a large-scale manufacturing process.

Another strategy to reduce the expression of harmful transgenes in the producer cells would be choosing a weaker promoter. For instance, by using p7.5 instead of the strong promoters mH5 or SSP helped to stabilize the measles virus fusion protein as transgene (24). However, this strategy also leads to reduced expression of the antigen upon vaccination (49). Importantly, as the transgene suppression system is artificially engineered into the CR pIX PRO suppressor cell line, antigen expression will - due to the absence of TetR and Plin-2 shRNA - not be suppressed in the vaccinees’ target cells upon administration of the rMVA.

There are also some limitations to our study. First, in concordance with Kremer et al. (38), we have not used a defined MOI for each passage of the passaging experiment which might have biased our results to an even accelerated negative selection of the rMVA. Furthermore, our results regarding M-DelIII stability in a CR19 background may not be completely translatable to conventional MVA since CR19 underwent a recombination that duplicated the genes MVA167 to the right ITR (30, 33). This might influence the proximal DelIII locus and also explain the appearance of large deletions in the context of the DelIII locus that have not been reported previously (23, 26, 27). On the other hand, it was shown that CR19 containing GFP and mCherry as a dual expression cassette in the DelIII locus is stable for at least 20 passages (33). Others used a genetically engineered and restructured DelIII site for integration of their inserts by removing non-essential MVA164R, 165R and 166R. In that case the transgene is integrated between the MVA163R (A50R) and MVA167R (B1R) to enhance the stability of the modified DelIII integration site in MVA (50). The obviously non-essential character of MVA165R and MVA166R flanking our demanding transgene in MVA-CR19 may favor larger deletions in DelIII and concomitant loss of transgene expression.

In summary, by suppressing the transgene expression we could obtain sustained transgene maintenance of an otherwise instable antigen, MfPV3 Ii-E1E2E6E7, for a considerable number of passages. The system was further improved by integration of the transgene into the more stable TK locus. This confirms that knock-down of transgene expression during MVA expansion can enhance virus yield, genetic stability, and transgene expression levels.

These data emphasize the importance of rigorous quality controls regarding correctness of the expression cassette’s sequence and protein production levels during all steps of rMVA generation and production. This should comprise characterization of bulk virus batches but also methods that allow detection of minority mrMVA populations, such as analysis of sufficient numbers of foci or deep-sequencing of the MVA population. As demonstrated herein, depending on the transgene, mutations may occur quickly and vary in nature depending to the transgene integration site. The novel MVA-adapted transgene expression suppression system can be employed to rescue the generation of otherwise hard-to-generate rMVA.

## Data availability statement

The original contributions presented in the study are included in the article/**Supplementary Material**. Further inquiries can be directed to the corresponding author.

## Author contributions

PN: Conceptualization, Data curation, Formal analysis, Investigation, Methodology, Visualization, Writing – original draft, Writing – review & editing. MM: Investigation, Writing – review & editing. MB: Methodology, Writing – review & editing. AK: Formal analysis, Investigation, Methodology, Writing – review & editing. DB: Writing – review & editing. CT: Funding acquisition, Writing – review & editing. PH: Funding acquisition, Writing – review & editing. IJ: Conceptualization, Formal analysis, Investigation, Methodology, Writing – review & editing. VS: Conceptualization, Formal analysis, Methodology, Writing – review & editing. BA: Formal analysis, Methodology, Writing – review & editing. RW: Conceptualization, Formal analysis, Funding acquisition, Methodology, Project administration, Resources, Supervision, Writing – review & editing.
