# Supplementary material for: Transgene expression knock-down in recombinant Modified Vaccinia virus Ankara vectors improves genetic stability and sustained transgene maintenance across multiple passages
Source: Front Immunol. 2024 Feb 6;15:1338492. doi: 10.3389/fimmu.2024.1338492 (PMC10877035; doi:10.3389/fimmu.2024.1338492)
Supplement: Supplementary file 1 [file DataSheet_1.pdf]

Supplementary Material

1 Supplementary Figures and Tables

1.1 Supplementary Figures

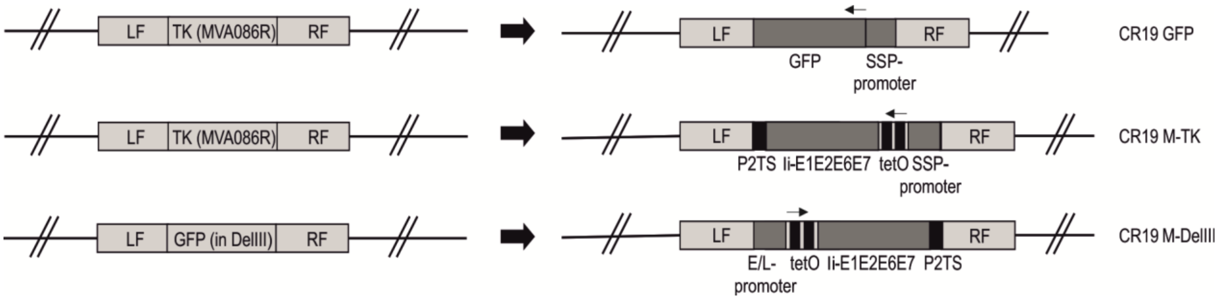

Supplementary Figure 1. Generation of rMVA. Schematic representation of the generated rMVA. Abbreviations: RF: right flank, LF: left flank, TK: thymidine kinase locus (MVA086R), GFP: green fluorescent protein, SSP-promoter: short synthetic promoter, tetO: tetracyclin operator, P2TS: Plin2 target sequence, DelIII: Deletion site III. Arrow indicates orf reading direction.

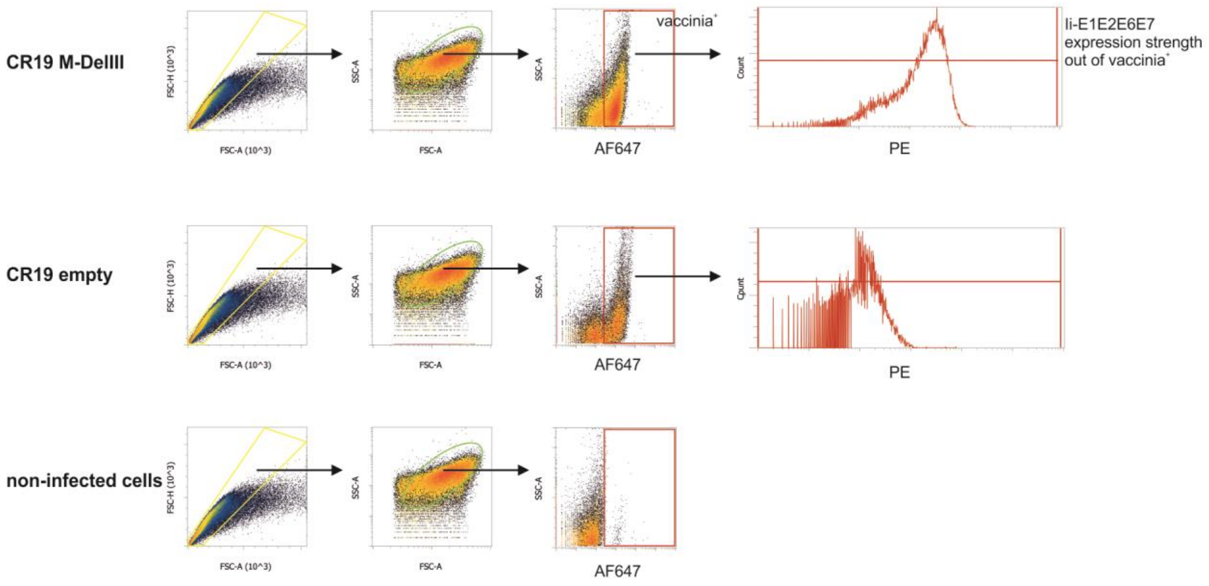

Supplementary Figure 2. Gating strategy to analyze transgene expression strength. Cells were gated on single cells using forward and side scatter. Vaccinia-infected cells (via rabbit anti-vaccinia and goat anti-rabbit AF647) were gated using non-infected CR pIX cells. MFI of Ii-E1E2E6E7 (via mouse anti-myc and goat anti-mouse PE) was measured from vaccinia-positive cells.

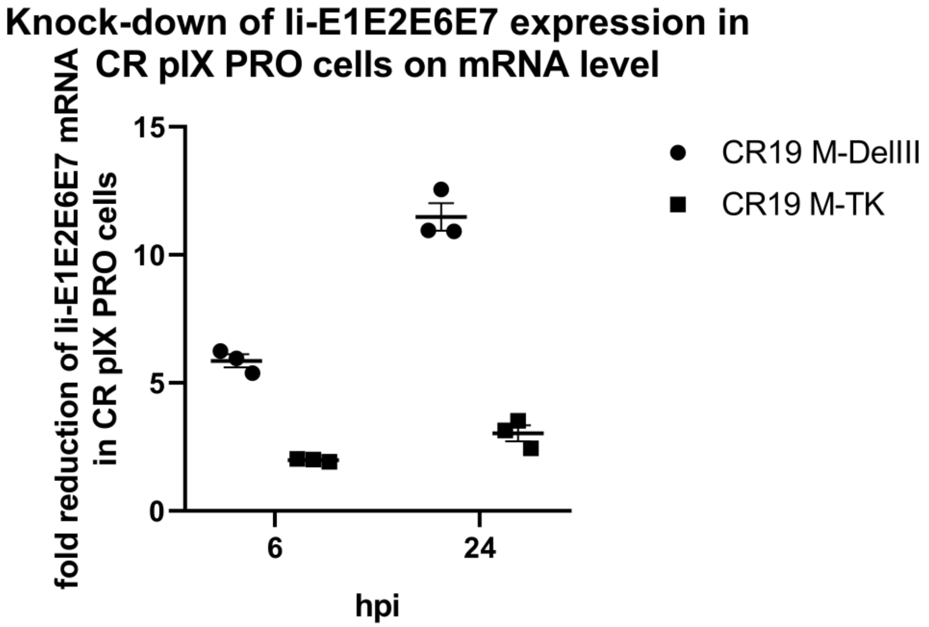

Supplementary Figure 3. Quantification of Ii-E1E2E6E7 transcript knock-down with RT-qPCR. Depicted is the fold reduction of Ii-E1E2E6E7 transcript level in CR pIX PRO suppressor cells compared to parental CR pIX cells of CR19 M-DelIII and CR19 M-TK after 6 and 24 hpi measured by RT-qPCR. Relative comparison of transcript levels between cell lines was normalized with the housekeeping gene MVA128L and calculated with the Pfaffl method. N=3 biological replicates, bars represent mean with SEM.

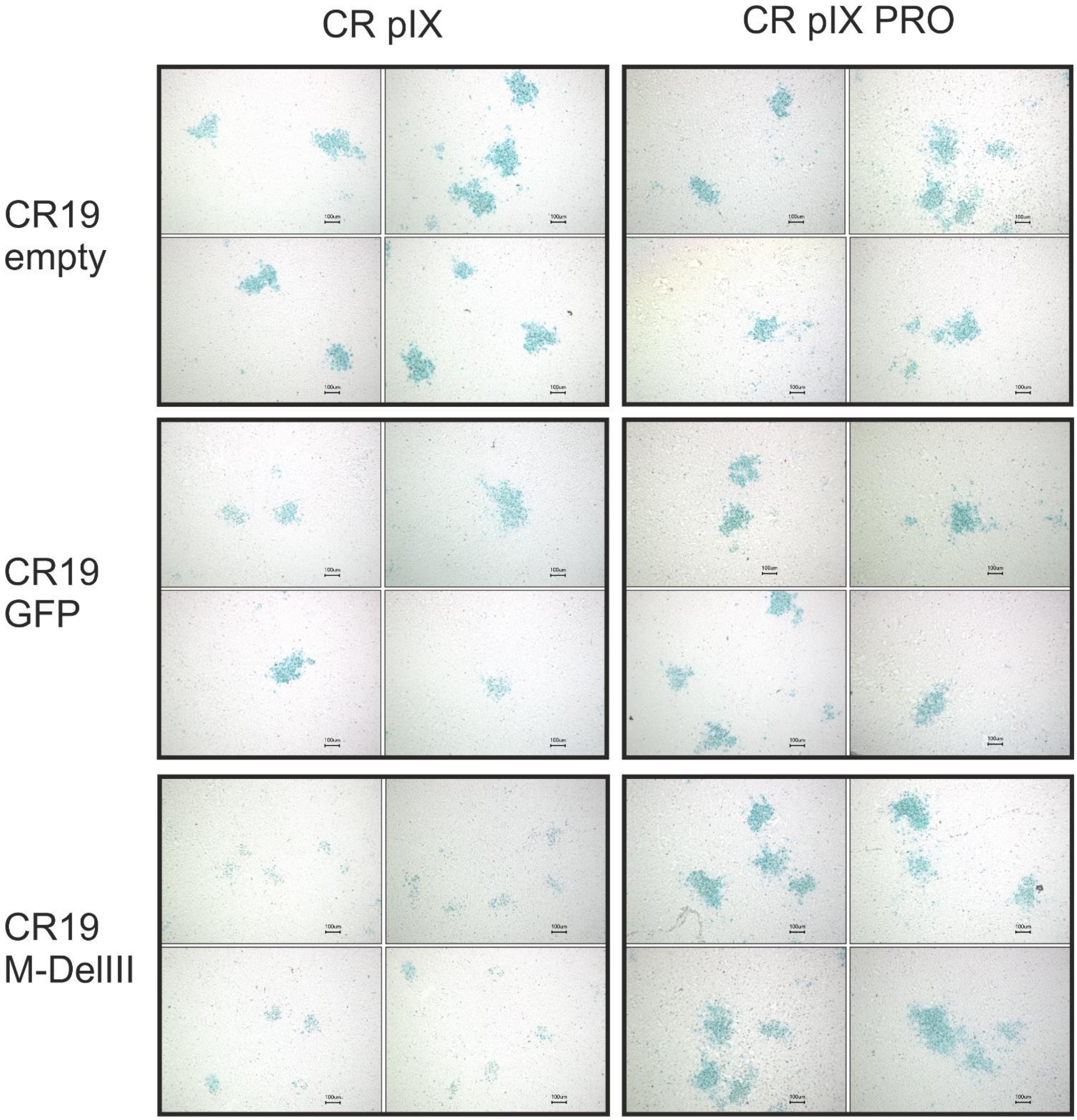

Supplementary Figure 4. CR pIX and CR pIX PRO suppressor cells were infected at an MOI of 0.01 with the indicated rMVA-CR19. 48 hpi, cells were fixed and stained with an anti-vaccinia antibody, an HRP-coupled secondary antibody and KPL Trublue substrate. Photos were taken on a Keyence inverted microscope at a magnification of 10. Bar represents 100 µm. For reference, the upper left image of each condition represents the same image as shown in **Figure 2B**.

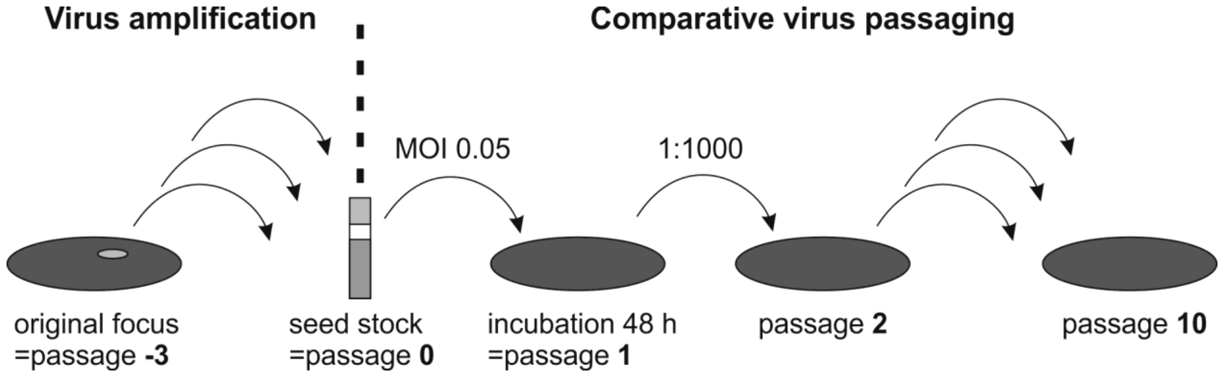

Supplementary Figure 5. Schematic representation of comparative passaging on parental CR pIX and CR pIX PRO suppressor cell lines. Initially, 3 expansion rounds on CR pIX PRO suppressor cells were needed to generate both seed stocks from the original focus-purified rMVA. Passaging was conducted from virus seed stocks of CR19 M-DelIII and CR19 M-TK (defined as passage 0). CR pIX and CR pIX PRO cells were infected with an MOI of 0.05 and incubated for 48 h. Cells were harvested with supernatant (passage 1), freeze-thawed for virus release and a dilution of 1:1000 was reinfected on CR pIX or CR pIX PRO cells, respectively. This was repeated until 10 passages were obtained.

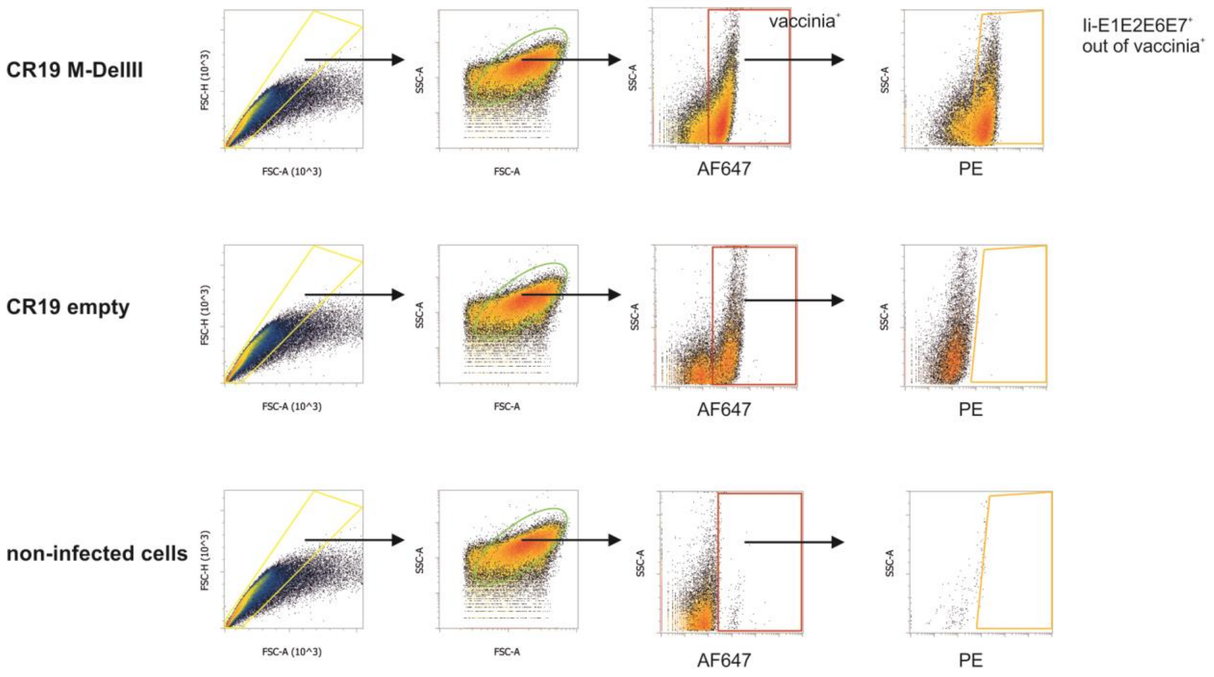

Supplementary Figure 6. Gating strategy to analyze the fraction of transgene-expressing MVA-infected cells. Cells were gated on single cells using forward and side scatter. Vaccinia-infected cells (via rabbit anti-vaccinia and goat anti-rabbit AF647) were gated using non-infected CR pIX cells. Vaccinia^{+} cells were then gated for Ii-E1E2E6E7 expression (via mouse anti-myc and goat anti-mouse PE) based on CR19-empty-infected control cells.

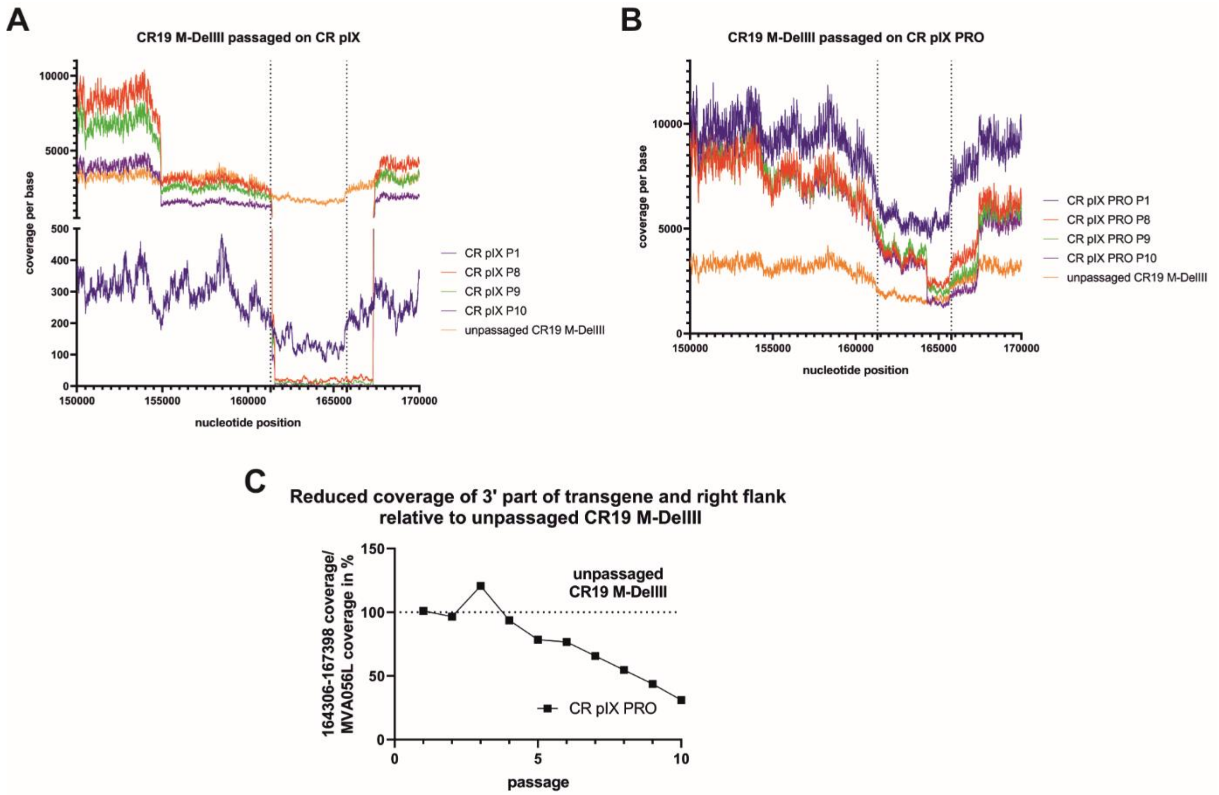

Supplementary Figure 7. Coverage maps and relative coverage of selected CR19 M-DelIII-derived samples. Depicted is the read coverage per nucleotide from the nucleotide position 150000 to 170000 of CR19 M-DelIII passaged on parental CR pIX (**A**) and CR pIX PRO suppressor (**B**) cells from selected passages. Dotted lines represent the position of the transgene (nucleotide position 161317 to 165771). (**C**) Depicted is the quotient of the mean read coverage of nucleotide position 164306 to 167398 (3’ part of Ii-E1E2E6E7 and right flank) and the mean read coverage of the essential MVA056L gene (MVA DNA polymerase, E9L) of CR19 M-DelIII passaged on CR pIX PRO cells, normalized to the unpassaged rMVA. Dashed line represents normalized read coverage of unpassaged rMVA used as starting material for passaging on both cell lines.

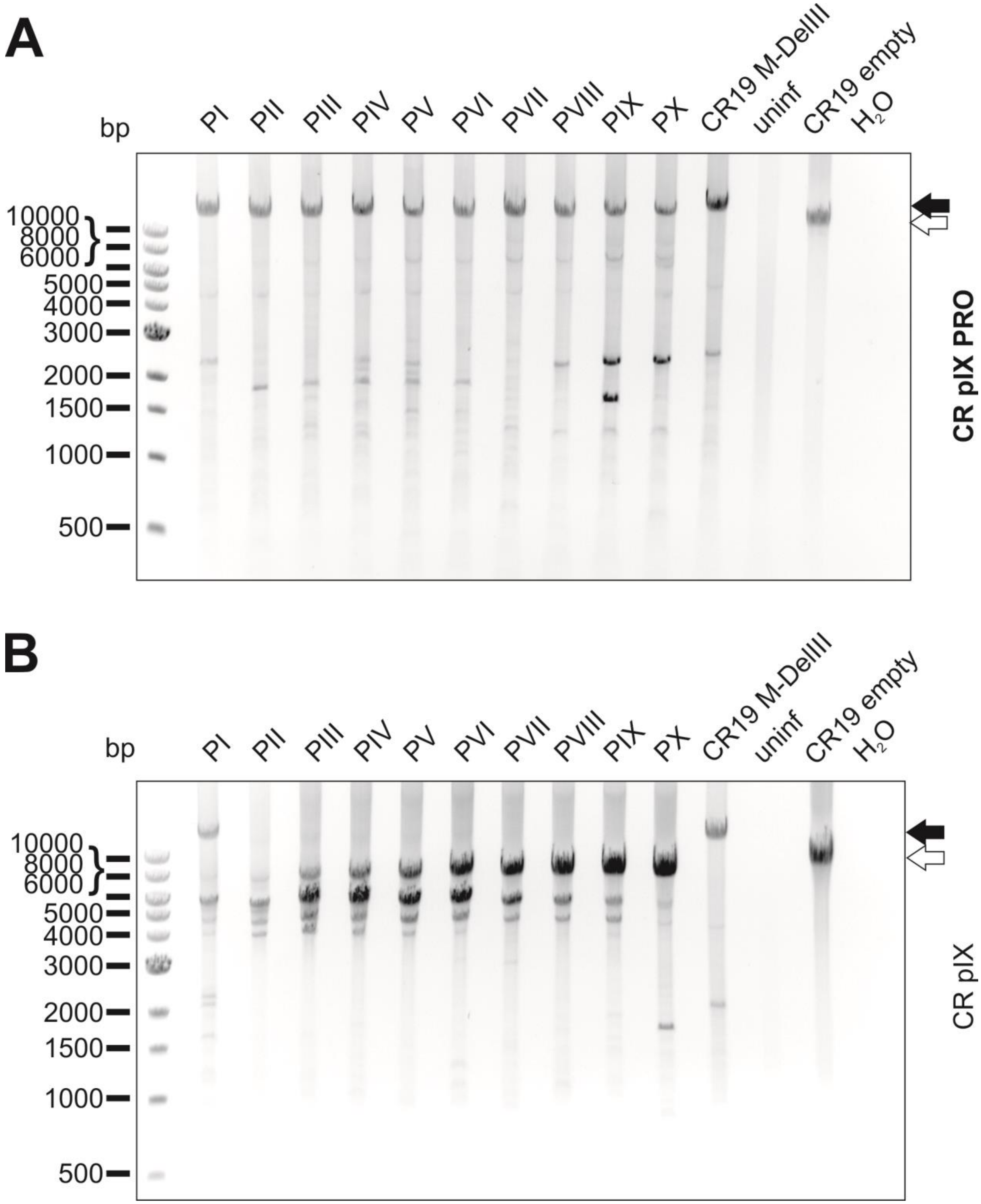

Supplementary Figure 8. Genotyping of CR19 M-DelIII passaged on CR pIX PRO suppressor (**A**) and parental CR pIX (**B**) cells by agarose gel analysis of PCR products obtained with the primer pair wide-III3-f and wide-III2-r. Expected size of CR19 M-DelIII: 14169 bp (black arrow); expected size of CR19 empty: 9471 bp (white arrow).

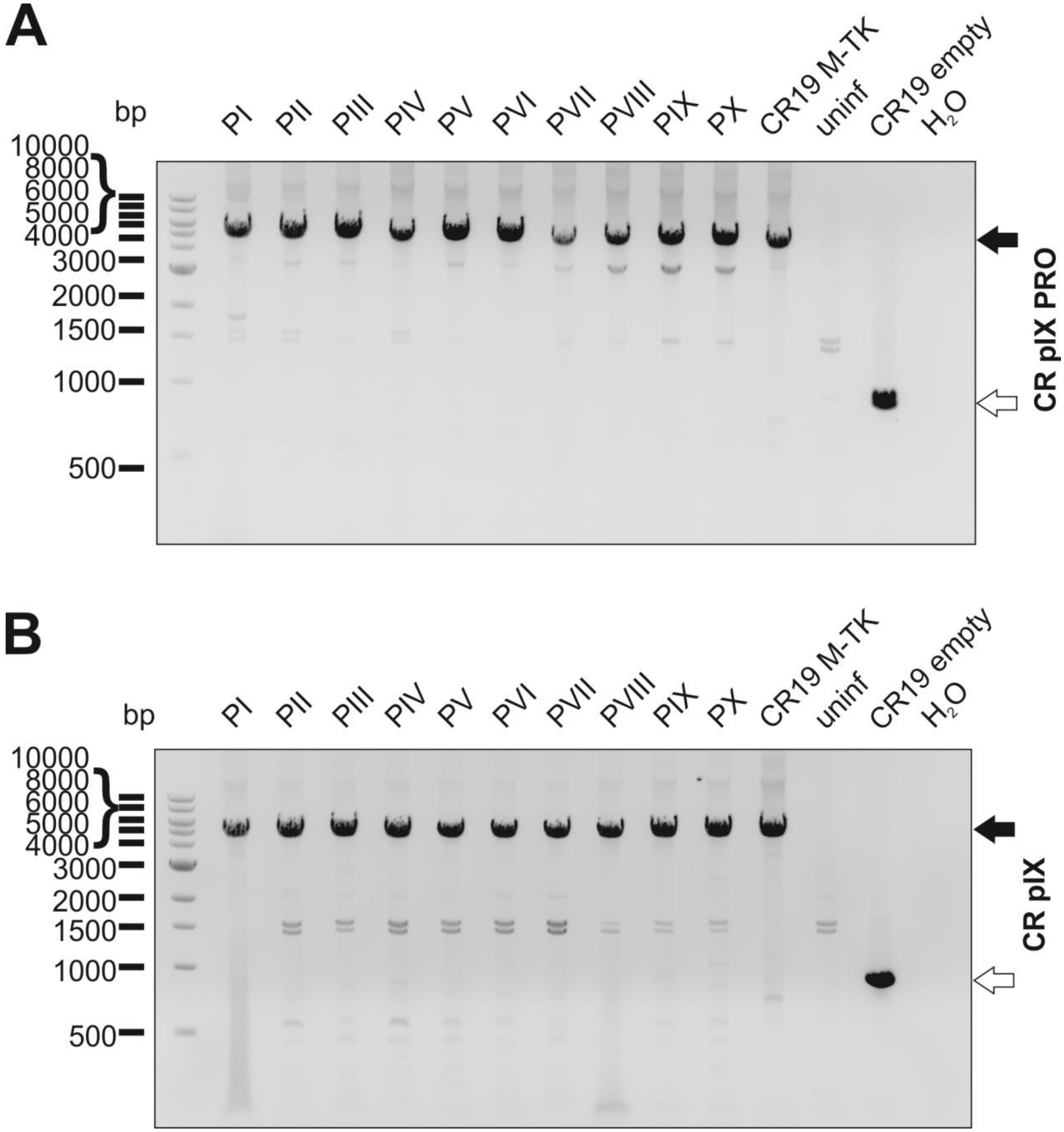

Supplementary Figure 9. Genotyping of CR19 M-TK passaged on CR pIX PRO suppressor (**A**) and parental CR pIX (**B**) cells by agarose gel analysis of PCR products obtained with the primer pair TK f and TK r. Expected size of CR19 M-TK of 5094 bp (black arrow) and expected size of CR19 empty of 920 bp (white arrow).

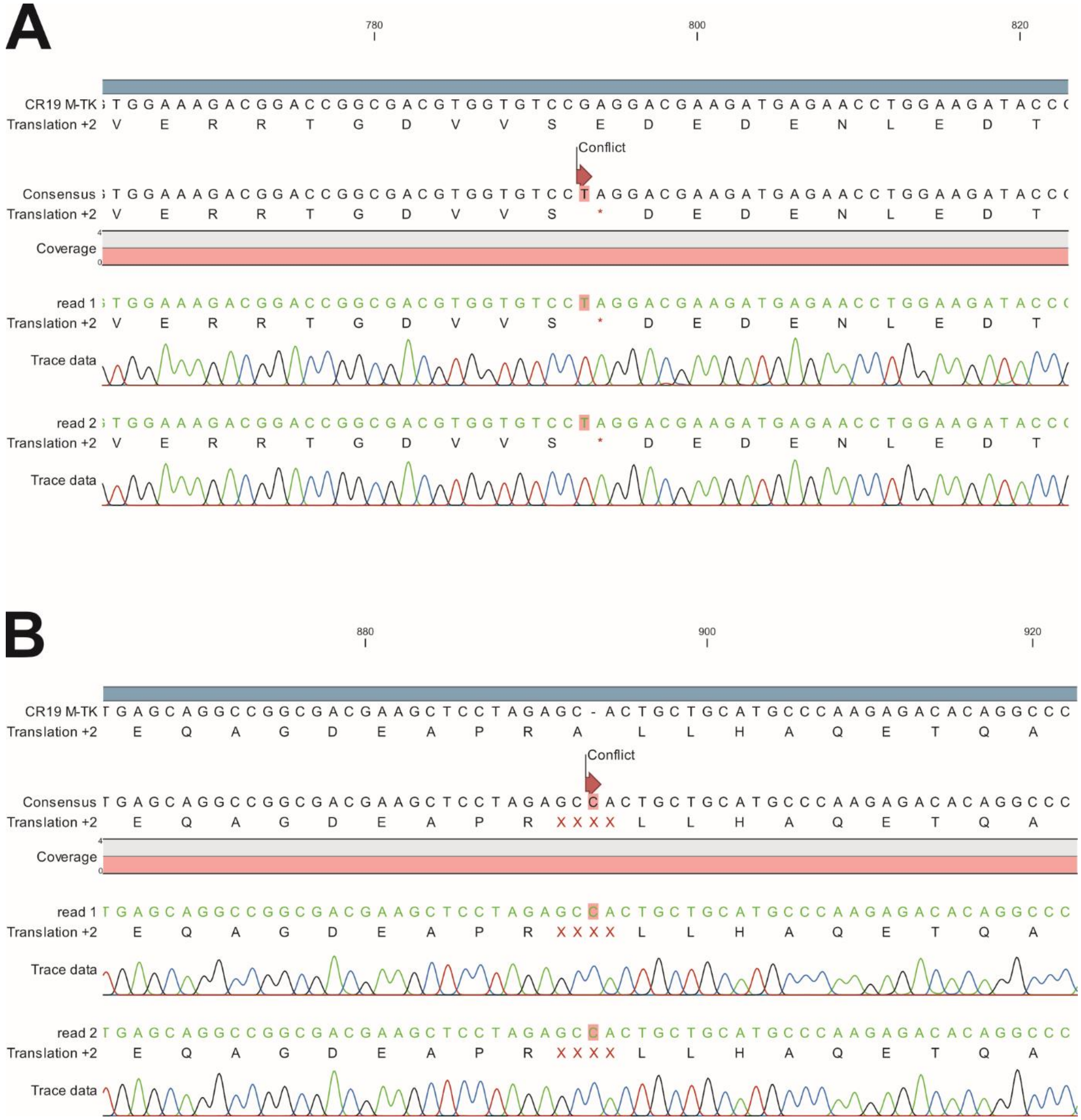

Supplementary Figure 10. Sanger sequencing on pJET1.2/blunt-subcloned PCR amplicons of passaged CR19 M-TK. The gDNA was prepared from bulk material of passage 3 (**A**) or passage 10 (**B**) and used as template in a PCR using the primer pair TK f and TK r flanking the transgene within the TK locus. The amplicons were cloned into pJET1.2/blunt and Sanger-sequenced. Both mutations E265*(**A**) and L299Tfs342* (**B**) could be observed when the sequencing results were aligned with the transgene.

1.2 Supplementary Tables

Supplementary Table 1: PCR analysis of focus isolated rMVA

Virus	passage	CR pIX PRO		CR pIX	
		transgene	DelVI	transgene	DelVI
CR19 M-DelIII	0	20*	20	13	20
	3	19	20	3	20
	10	18	20	2	20
CR19 M-TK	0	20	20	20	20
	3	20	20	18	20
	10	20	20	20	20

*numbers indicate the number of focus isolated rMVA, which showed an amplicon at the expected size. For each condition 20 focus-isolated rMVA were tested. DelVI served as sampling control.

Supplementary Table 2: coordinates of rMVA gene and transgene locations (based on KY633487.1)

Virus	Gene	5’ start	3’ end
CR19 M-DelIII	Ii-E1E2E6E7	161317	165771
	DelIII locus incl. flanks	160201	166525
	MVA056L	63330	60310
	Whole genome	1	194409
CR19 M-TK	Ii-E1E2E6E7	91871	87417
	TK locus incl. flanks	92734	86851
	MVA056L	63330	60310
	Whole genome	1	193884
